# Effects of *Bacillus coagulans* (GBI-30, 6086) Supplementation on the Fecal Characteristics and Microbiota of Healthy Adult Dogs Subjected to an Abrupt Diet Change

**DOI:** 10.3390/microorganisms13112462

**Published:** 2025-10-28

**Authors:** Sofia M. Wilson, Yifei Kang, Jocelyn F. Wren, John F. Menton, Elena Vinay, Mathieu Millette, Melissa R. Kelly, Kelly S. Swanson

**Affiliations:** 1 Department of Animal Sciences, University of Illinois Urbana-Champaign, Urbana, IL 61801, USAjfwren2@illinois.edu (J.F.W.); 2 The Carl R. Woese Institute for Genomic Biology, University of Illinois Urbana-Champaign, Urbana, IL 61801, USA; 3 Kerry Group, Beloit, WI 53511, USA; 4 Kerry (Canada), Laval, QC H7V 4B3, Canada; mathieu.millette@kerry.com; 5 Science Made Simple, LLC, Winston Salem, NC 27101, USA; 6 Department of Veterinary Medicine, University of Illinois Urbana-Champaign, Urbana, IL 61802, USA; 7 Division of Nutritional Sciences, University of Illinois Urbana-Champaign, Urbana, IL 61801, USA

**Keywords:** canine microbiota, gastrointestinal health, probiotic, nutrition, *Bacillus coagulans*

## Abstract

Studies in humans and livestock have demonstrated *Bacillus coagulans* GBI-30, 6086 to have probiotic potential, suggesting that it may alleviate gastrointestinal (GI) distress commonly associated with diet change in dogs. This study aimed to evaluate the effects of *B. coagulans* GBI-30, 6086 on fecal scores, pH, dry matter (DM) percentage, and microbiota populations of dogs following an abrupt diet change. English Pointer dogs (*n* = 12; age = 5.9 ± 2.5 yr; body weight = 26.6 ± 6.1 kg) were used in a replicated 3 × 3 Latin square design and fed commercial diets containing no probiotics or prebiotics. The following treatments were administered orally in gelatin capsules before each daily feeding: (1) placebo control (250 mg maltodextrin/day); (2) *B. coagulans* [low dose; 5 × 10^8^ colony-forming units (CFU)/day]; and (3) *B. coagulans* (high dose; 2.5 × 10^9^ CFU/day). An extruded kibble diet was fed for 28 days. Dogs were then abruptly switched to a canned diet and fed for 14 days, with fecal samples collected before and 2, 6, 10, and 14 days after diet change. All data were analyzed using the Mixed Models procedure of SAS 9.4, testing the effects of treatment, time, and treatment*time interactions. Treatment*time interactions were not observed, but the abrupt diet change reduced (*p* < 0.0001) fecal DM content, increased (*p* < 0.0001) fecal scores and pH, and reduced (*p* < 0.0001) fecal bacterial species richness and phylogenetic diversity. Diet change also increased (*p* < 0.001) fecal Bacteroidota, Fusobacteriota, and Proteobacteria, decreased (*p* < 0.001) fecal Firmicutes, and altered ~40 fecal bacterial genera relative abundances. Diet-induced changes were minimally impacted by *B. coagulans*, but fecal scores tended to be lower (i.e., firmer stools; *p* < 0.10), fecal *E. coli* and *Faecalibacterium* abundances were greater (*p* < 0.05), and fecal bacterial phylogenetic diversity was higher (*p* < 0.05) in dogs supplemented with the low dose than in controls. Our results demonstrate that abruptly transitioning dogs from a kibble to a canned diet negatively influences fecal characteristics and considerably shifts the composition of the fecal microbiota. Supplementation with *B. coagulans* did not mitigate the diet-induced shifts to fecal characteristics and most of the microbial taxa, although the low dose impacted some microbial taxa. Further investigation into optimal inclusion levels in pet foods is warranted.

## 1. Introduction

In a previous study, supplementing the bacterial strain *B. coagulans* GBI-30, 6086 to healthy dogs slightly improved fecal scores and did not negatively impact nutrient digestibility or canine health outcomes, although tangible benefits to host health and the fecal microbiota were limited [1]. Research has demonstrated the successful use of probiotics in dogs on a variety of health outcomes, but most of these studies were conducted in populations with underlying disease [2,3]. Evidence supporting the health-promoting effects of probiotics in healthy individuals is less consistent [4] despite increased demand for probiotics among pet owners, likely due to the promotion of these products to the broader consumer market rather than for use with specific health conditions. It can be challenging to demonstrate reproducible positive physiological effects in healthy populations when functional impairments are absent [5], especially when considering the natural biological variation of living subjects.

However, the intestinal microbiome has been shown to respond rapidly to dietary composition regardless of health status [6,7,8], affecting microbial contributions to host health, such as nutrient metabolism, production of fermentative metabolites, regulation of gut barrier function, and modulation of the immune system [9]. Considering that probiotic microorganisms benefit host health when supplemented in adequate amounts [10], they may have the potential to support the (gastrointestinal) GI microbiota and reduce GI distress associated with dietary change. Functional ingredients such as dietary fiber [8], yeast cell wall fraction [11], and live yeast [12] have previously exhibited the ability to modulate microbial functions, improve immune function, and mitigate diet-induced dysbiosis in dogs undergoing diet transition, but there is limited research in this area pertaining to probiotics.

To date, several *Bacillus* spp. have been evaluated for their probiotic potential [13]. As spore-formers, *Bacillus* spp. are distinguished by their ability to withstand commercial processing and unfavorable GI conditions, such as extreme temperature and pH ranges, high bile salt concentrations, high pressure, dehydration, and long storage [13,14,15,16]. These characteristics may lend resiliency to *Bacillus* spp. during a diet transition, wherein changes to macronutrient content alter the intestinal environment [8,17]. *Bacillus* spp. spores have been shown to play a role in gut-associated lymphoid tissue development and host immune responses [18], while metabolically active vegetative cells produce a host of antimicrobial compounds [19] and enzymes [20,21] that influence nutrient metabolism and regulation of commensal bacteria.

The species, *Bacillus coagulans*, is now known as *Heyndrickxia coagulans* and *Weizmannia coagulans* [22,23,24], although most products continue to use the common name *Bacillus*. *B. coagulans* has previously been reported to modulate GI microbiota, host immunity, and metabolism in monogastric mammals [25,26,27,28,29], although microbial metabolism is strain-specific and dose-dependent. There are currently few studies investigating *B. coagulans* as a single-strain probiotic in dogs. One group reported increased nutrient digestibility in dogs fed a diet coated with *B. coagulans* GBI-30, 6086 (1 × 10^9^ CFU/d) [30] but without changes to fecal characteristics and metabolites. Our previous study demonstrated that *B. coagulans* GBI-30, 6086 (5 × 10^8^ CFU/d) improved fecal scores and increased the relative abundance of some beneficial fecal bacteria (e.g., *Faecalibacterium*) without negatively impacting nutrient digestibility or canine health, although benefits to host health were modest [1]. In the current study, we aimed to evaluate the effects of two dosage levels of *B. coagulans* GBI-30, 6086 (low dose 5 × 10^8^ CFU/d; high dose 2.5 × 10^9^ CFU/d) on the fecal scores, pH, dry matter (DM) percentage, and microbiota populations of healthy adult dogs following an abrupt diet change. We hypothesized that the abrupt diet change would alter the fecal characteristics and shift fecal microbiota populations. We also hypothesized that *B. coagulans* consumption would attenuate the changes to the fecal characteristics and shifts to the fecal microbiota, with greater efficacy observed in dogs given the high dose vs. the low dose.

## 2. Materials and Methods

Animals: A replicated 3 × 3 Latin square design experiment was conducted. Twelve healthy adult English Pointer dogs were used (6 intact males; 6 intact females; age = 5.9 ± 2.5 yr; BW = 26.6 ± 6.1 kg). All dogs were housed individually (inside run = 1.17 m × 1.42 m; outside run = 1.08 m × 3.05 m) at Kennelwood, Inc. (Champaign, IL, USA). Dogs had ad libitum access to fresh water and were fed once daily to maintain BW. The amount of food offered was based on previous feeding records and the estimated caloric content of the diets fed. Daily food intake was recorded, and any refused food was measured each day to calculate intake. Dogs were weighed, and body condition scores were assessed using a 9-point scale [31] once a week prior to feeding.

Experimental timeline and treatments: This study was composed of three 42-day periods. Each experimental period consisted of a 28-day adaptation phase and a 14-day fecal collection phase. A commercial diet containing no probiotics or prebiotics and little fermentable fiber (Best Dog Diet 21:12; Mid-South Feeds Inc., Alma, GA, USA) was fed to all dogs during the 28-day diet transition phase. On day 28 (after fecal samples were collected), dogs were abruptly changed to a canned diet (Pedigree Chopped Ground Dinner with Chicken Adult Canned Dog Food; Mars Petcare US, Franklin, TN, USA). All dogs were fed the canned diet for 14 days, with fecal samples being scored and analyzed for pH, DM content, and microbiota before and 2, 6, 10, and 14 days after diet change. Both the kibble and canned diets were formulated to meet all Association of American Feed Control Officials [32] nutrient recommendations for adult dogs at maintenance. The chemical compositions of the commercial diets analyzed are summarized in Table 1. Probiotic spores (*Bacillus coagulans* GBI-30,166 6086; BC30, Kerry, Inc. Beloit, WI, USA) and placebo capsules were administered orally in gelatin capsules before each daily feeding, with the following treatments tested: (1) placebo control (250 mg maltodextrin/day); (2) *B. coagulans* GBI-30, 6086 (low dose; 5 × 10^8^ CFU/day); and (3) *B. coagulans* GBI-30, 6086 (high dose; 2.5 × 10^9^ CFU/day). *B. coagulans* GBI-30, 6086 (ATCC PTA-6086; Manassas, VA, USA) is a variant of the type strain *B. coagulans* Hammer (ATCC 7050; DSM1; Manassas, VA, USA), which was isolated from spoiled canned milk.

Fecal collection, scoring, and analysis: During the fecal collection phase, one fresh fecal sample (within 15 min of defecation) was scored and collected for measurement of pH, DM content, and microbiota populations. Fecal scores were determined according to the Waltham feces scoring system [33] using the following scale: 1 = hard dry and crumbly; 1.5 = hard and dry; 2 = well formed; does not leave a mark when picked up; “kickable”; 2.5 = well formed, with a slightly moist surface, which leaves a mark when picked up; 3 = moist beginning to lose form, leaving a definite mark when picked up; 3.5 = very moist, but still has some definite form; 4 = the majority, if not all the form is lost; poor consistency; viscous; 4.5 = diarrhea, with some areas of consistency; 5 = watery diarrhea. Fecal pH was measured immediately using an AP10 pH meter (Denver Instrument, Bohemia, NY, USA) equipped with a Beckman Electrode (Beckman Instruments Inc., Fullerton, CA, USA). After pH was measured, an aliquot was collected for DM determination in accordance with AOAC [34] using a 105 °C oven. An aliquot was collected into a sterile cryogenic vial (DWK Life Sciences, Millville, NJ, USA), immediately frozen in dry ice, and stored at −80 °C until analysis for fecal bacterial DNA extraction.

Dietary chemical analyses: Diet subsamples were collected from each opened bag, frozen at −20 °C until the completion of the study, then pooled. One can of canned diet was spared each day during the abrupt diet transition phase, with subsamples pooled together at the end of the study. Before analysis, canned diets were lyophilized (Dura-Dry MP microprocessor-controlled freeze-dryer, FTS Systems, Stone Ridge, NY, USA). Diet samples were ground in a Wiley mill (model 4, Thomas Scientific, Swedesboro, NJ, USA) through a 2 mm screen and then analyzed for DM and ash according to AOAC (2006; methods 934.01 and 942.05), with organic matter (OM) calculated. Crude protein (CP) was calculated from Leco (TruMac N, Leco Corporation, St. Joseph, MI, USA) total nitrogen values according to AOAC ([34]; method 992.15). Total lipid content (acid-hydrolyzed fat; AHF) was determined according to the methods of the American Association of Cereal Chemists [35] and Budde [36]. Total dietary fiber (TDF) content of diets was determined according to Prosky et al. [37]. Gross energy was measured using an oxygen bomb calorimeter (model 6200, Parr Instruments, Moline, IL, USA).

Fecal DNA extraction and PacBio sequencing of 16S rRNA gene amplicons: Bacterial DNA from fecal samples was extracted according to the manufacturer’s instructions using the DNeasy PowerLyzer PowerSoil Kit (MoBio Laboratories, Carlsbad, CA, USA) with bead beating using a vortex adaptor. Concentrations of extracted DNA were quantified using a Qubit 3.0 Fluorometer (Life Technologies, Grand Island, NY, USA). The quality of extracted DNA was assessed by electrophoresis using agarose gels (E-Gel EX Gel 1%; Invitrogen, Carlsbad, CA, USA). The Roy J. Carver Biotechnology Center at the University of Illinois performed PacBio sequencing. The 16S rRNA gene amplicons were generated with the barcoded full-length 16S rRNA gene primers from PacBio and the 2× Roche KAPA HiFi Hot Start Ready Mix (Roche, Wilmington, MA, USA). Full-length 16S rRNA gene PacBio (Pacific Biology, Menlo Park, CA, USA) primers (forward: AGRGTTYGATYMTGGCTCAG; reverse: RGYTACCTTGTTACGACTT) were added in accordance with the PacBio protocol. The amplicons were pooled and converted to a library with the SMRT Bell Express Template Prep kit 3.0. (Pacific Biology, Menlo Park, CA, USA). The library was sequenced on a SMRT cell 8M in the PacBio Sequel IIe using the CCS sequencing mode and a 15 h movie time. Analysis of CCS was done using SMRT Link V11.1.0 using the following parameters: minimum passes 3, and minimum rq 0.999; HiFi presets (minimum score of 80; minimum end score of 50, minimum reference (read) span of 0.75); asymmetric (different, minimum number of scoring barcode regions 2).

Sequence data processing: PacBio-based FASTQ reads were processed using a Nextflow-based workflow, TADA [38]. TADA v1.1 automates using DADA2 v1.22 [39] for trimming and denoising reads based on the protocols used for PacBio data to generate amplicon sequence variants. The input sample sheet was a simple comma-separated file with the sample ID and the path for the relevant sample FASTQ file. The DADA2 implementation of the RDP classifier [40] was used to classify reads using the SILVA 138.1 release (23 February 2024), with a database formatted for PacBio HiFi read data (https://zenodo.org/record/4587955). Multiple sequence alignment and maximum likelihood phylogenetic analysis were performed using DECIPHER v2.22 [41] and Fasttree v2.1.10 [42]. Alpha diversity was evaluated using Observed Features (richness), Chao1 (richness estimator accounting for unseen taxa), Faith’s PD (phylogenetic branch length), and Simpson/Inverse Simpson indices (dominance and diversity). Beta diversity was assessed using Bray–Curtis dissimilarity (abundance-based) and UniFrac distances, with unweighted metrics reflecting presence/absence and weighted metrics incorporating relative abundances along phylogenetic lineages.

Quantitative polymerase chain reaction (qPCR): DNA was extracted from an aliquot of 100–120 mg fecal sample using a bead-beating method with a MO BIO Power Soil DNA isolation kit (Qiagen, Carlsbad, CA, USA). The qPCR assays were applied to quantify total bacteria, *Blautia*, *Clostridium (Peptacetobacter) hiranonis*, *Escherichia coli*, *Faecalibacterium*, *Fusobacterium*, *Streptococcus*, and *Turicibacter*. The qPCR assays have been described previously [43]. Both positive and negative controls were included for all qPCR assays to ensure the accuracy and reliability of the results.

Statistical analyses: Data were analyzed using the Mixed Models procedure of SAS version 9.4 (SAS Institute, Inc., Cary, NC, USA), with the fixed effect of treatment and dog considered a random effect, for all analyses. The effects of treatment, time, and treatment*time were tested. Data were tested for normality using the UNIVARIATE procedure of SAS. If data did not meet normality, a logarithmic transformation was applied. If the transformation failed, data were analyzed using Kruskal–Wallis tests to determine significance. Differences between treatments were determined using a Fisher-protected least significant difference with a Tukey adjustment to control for experiment-wise error. Reported pooled standard errors of the means (SEMs) were determined according to the Mixed Models procedure of SAS. Data are reported as means ± pooled SEM, with statistical significance set as *p* < 0.05 and trends set as *p* < 0.10. For analysis of microbial relative abundance, R (version 4.3.2) was utilized, along with specific packages including dplyr, ggplot2, ggpubr, and FSA. For variables with an average of any group greater than 0.1%, Kruskal–Wallis tests were used to ascertain differences between treatments. Pairwise comparisons were performed using the Dwass, Steel, Critchlow–Fligner multiple-comparison analysis, with statistically significant differences (*p* < 0.05) and trends (*p* < 0.10) being identified. 

## 3. Results

**Figure 1:**
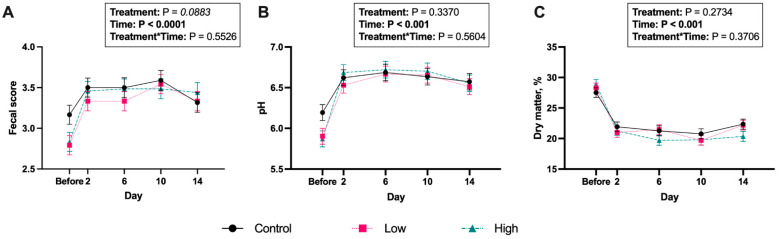
Fresh fecal characteristics, including fecal scores (**A**), fecal pH (**B**), and fecal dry matter percentage (**C**) of *Bacillus coagulans* GBI-30, 6086-supplemented dogs before and after an abrupt diet change. Samples were collected before and 2, 6, 10, and 14 d after change.

Before diet transition, average food intake for dogs eating the kibble diet was 595.3 g/d in placebo-supplemented dogs, 542.0 g/d in dogs supplemented with the low dose of *B. coagulans*, and 582.7 g/d in dogs fed the high dose of *B. coagulans*. Following the transition to a canned diet, average food intake was 1756.0 g/d in placebo-supplemented dogs, 1737.8 g/d in dogs supplemented with the low dose of *B. coagulans*, and 1780.4 g/d in dogs fed the high dose of *B. coagulans*. Food intake was not affected by *B. coagulans* supplementation before or after diet transition. The abrupt change to a canned diet reduced (*p* < 0.0001) fecal DM content and increased (*p* < 0.0001) fecal scores and pH (Figure 1; Supplementary File S1). Fecal pH and DM were not impacted by *B. coagulans* treatment, but fecal scores tended to be lower (i.e., firmer stools; *p* < 0.10) in dogs supplemented with the low dose of *B. coagulans* compared with controls.

**Figure 2:**
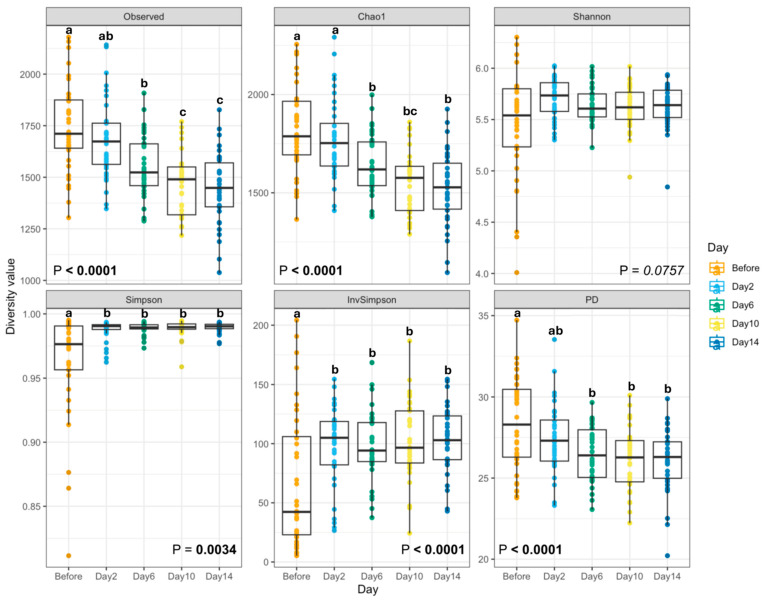
Fecal bacterial alpha diversity indices (by time) of dogs supplemented with *Bacillus coagulans* GBI-30, 6086, before and after an abrupt diet change. Pairwise comparisons between days (before, day 2, day 6, day 10, and day 14) were performed, with significant differences indicated with unlike superscript letters (*p* < 0.05).

**Figure 3:**
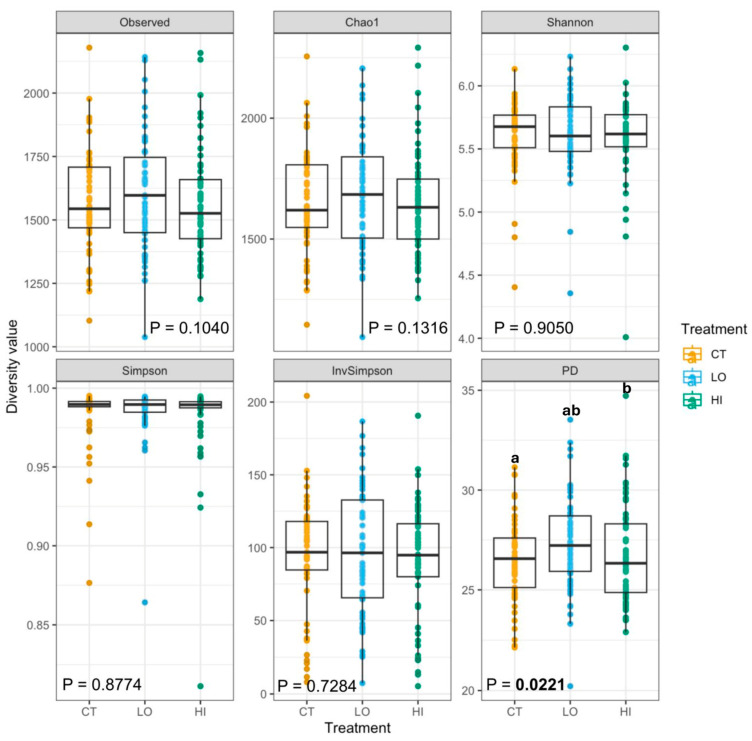
Fecal bacterial alpha diversity indices (by treatment) of dogs supplemented with *Bacillus coagulans* GBI-30, 6086, before and after an abrupt diet change. Significant differences are indicated with unlike superscript letters (*p* < 0.05).

After the dietary change, a few alpha diversity measures (Observed features, Chao1, and Faith’s PD) were reduced (*p* < 0.0001), while others (Simpson Index; Inverse Simpson Index) were increased (*p* < 0.01) (Figure 2). While species richness was markedly reduced, evenness remained stable or slightly increased, with no single species becoming overwhelmingly dominant. Collectively, these outcomes demonstrate that the change to the canned diet reduced fecal bacterial diversity, with notable reductions in richness and phylogenetic diversity following diet transition. *B. coagulans* treatment did not greatly affect fecal bacterial alpha diversity during the abrupt diet change. While one measure of fecal alpha diversity [Faith’s phylogenetic diversity (PD)] was higher (*p* < 0.05) in dogs supplemented with the low *B. coagulans* dose than those given the placebo (Figure 3), all other alpha diversity measures were unaffected by treatment.

**Figure 4:**
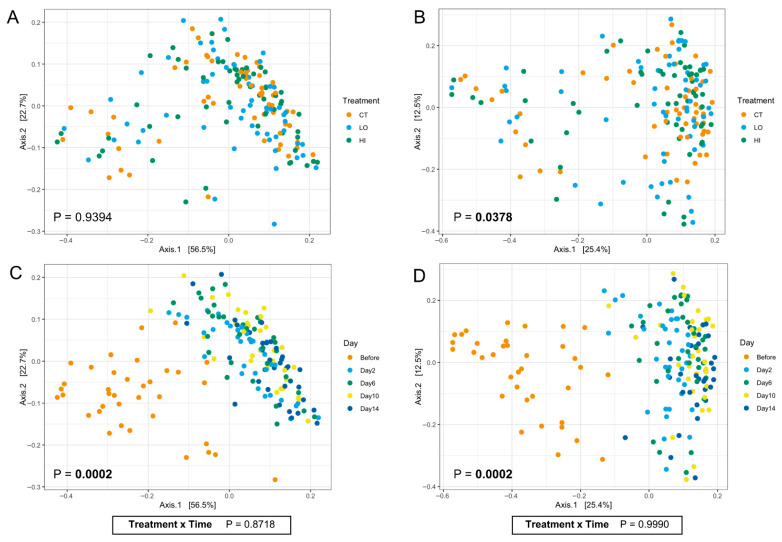
PCoA plots of fecal bacterial beta diversity indices, represented by weighted Unifrac (**A**/**C**) and by Bray–Curtis dissimilarity (**B**/**D**) distances, of dogs supplemented with *Bacillus coagulans* GBI-30, 6086 before and after an abrupt diet change.

Fecal bacterial beta diversity was greatly altered following the abrupt diet change (Figure 4A–D). Fecal samples collected prior to dietary change were different (*p* < 0.01) than samples collected at all other time points. According to weighted Unifrac phylogenetic metrics, fecal samples collected 2 days and 6 days after the diet change were also different (*p* < 0.05) from those collected 14 days after the diet change. According to Bray–Curtis dissimilarity metrics, fecal samples collected 2 days after diet change were different (*p* < 0.01) from those collected 10 and 14 days after diet change, and fecal samples collected 6 days after diet change were different (*p* < 0.05) from those collected 2 and 14 days after diet change. A treatment (*B. coagulans*) effect was observed (*p* < 0.05) for fecal beta diversity according to Bray–Curtis dissimilarity metrics, but did not differ after pairwise comparisons, indicating that while treatment influenced community structure overall, the differences between treatment groups were modest. This may have been driven by subtle, diffuse differences across groups and high within-group variability rather than strong separations between specific treatments.

**Figure 5:**
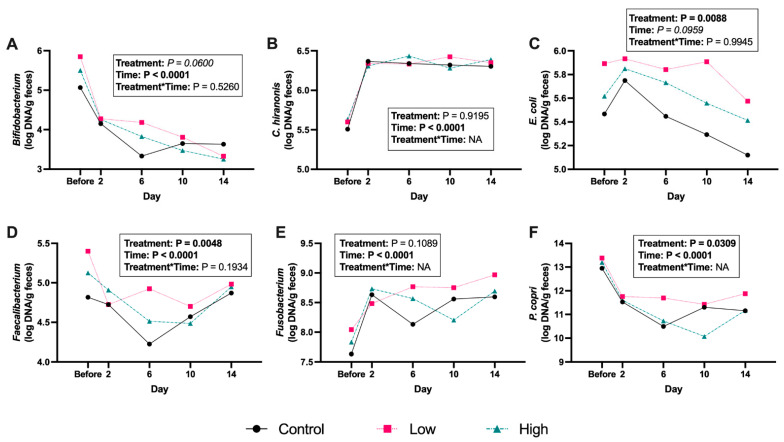
Fecal bacterial abundance (log DNA/gram feces) [*Bifidobacterium* (**A**), *C. hiranonis* (**B**), *E. coli* (**C**), *Faecalibacterium* (**D**), *Fusobacterium* (**E**), and *P. copri* (**F**)] of *Bacillus coagulans* GBI-30, 6086-supplemented dogs before and after an abrupt diet change. Samples were collected before and 2, 6, 10, and 14 d after change.

The abrupt change to a canned diet increased (*p* < 0.01) fecal *Clostridium hiranonis*, *Collinsella*, *Fusobacterium*, and *Ruminococcus gnavus* abundances and decreased (*p* < 0.01) fecal *Bifidobacterium*, *Blautia*, *Faecalibacterium*, *Prevotella copri*, *Streptococcus*, and *Turicibacter* abundances (Figure 5; Supplementary File S1). Fecal abundances were impacted by probiotic treatment, where *E. coli* and *Faecalibacterium* were greater (*p* < 0.05) in dogs supplemented with the low dose of *B. coagulans* compared to the control group. Fecal abundance of *P. copri* was higher (*p* < 0.05) in dogs supplemented with the low dose of *B. coagulans* compared to the high dose and control groups, but abundance only tended to differ between these treatments after multiple pairwise comparisons. Fecal abundances of *Bacteroides* and *Bifidobacterium* tended to be greater (*p* < 0.10) in dogs fed the low dose of *B. coagulans* compared to the control group. No treatment*time interactions were observed for bacterial abundances.

**Figure 6:**
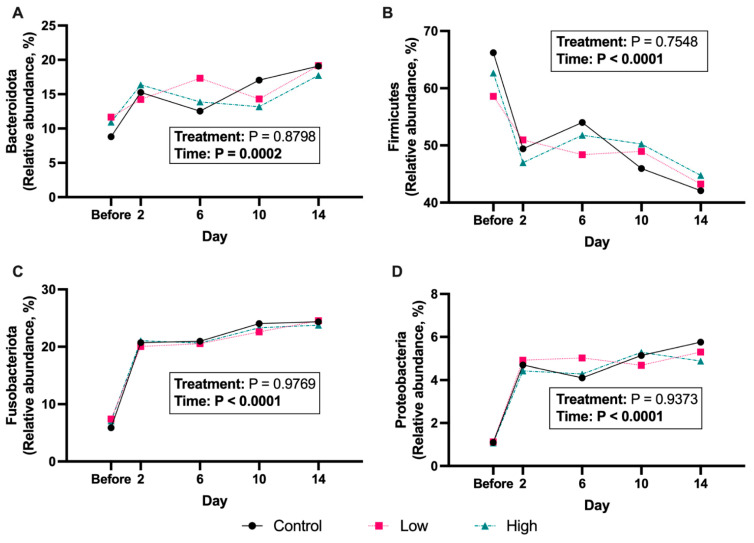
Relative abundances (% of sequences) of the main bacterial phyla [Bacteroidota (**A**), Firmicutes (**B**), Fusobacteriota (**C**), and Proteobacteria (**D**)] in feces of *Bacillus coagulans* GBI-30, 6086-supplemented dogs before and after an abrupt diet change. Samples were collected before and 2, 6, 10, and 14 d after change.

**Figure 7:**
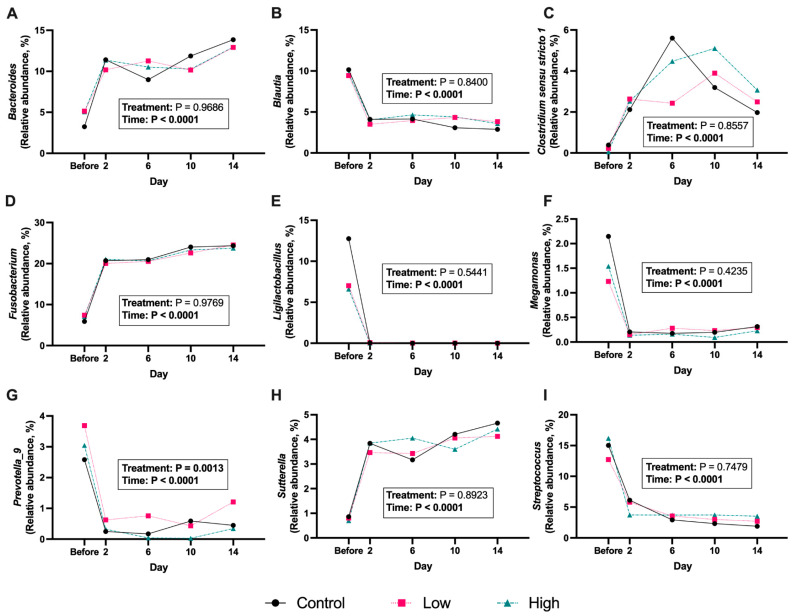
Relative abundances (% of sequences) of select bacterial genera [*Bacteroides* (**A**), *Blautia* (**B**), *Clostridium sensu stricto 1* (**C**), *Fusobacterium* (**D**), *Ligilactobacillus* (**E**), *Megamonas* (**F**), *Prevotella_9* (**G**), *Sutterella* (**H**), and *Streptococcus* (**I**)] in feces of *Bacillus coagulans* GBI-30, 6086-supplemented dogs before and after an abrupt diet change. Samples were collected before and 2, 6, 10, and 14 d after change.

The predominant fecal bacterial phyla present in all dogs of this study before and after diet change were Firmicutes (from 62.5 ± 3.11% to 43.4 ± 1.09% of sequences), Fusobacteriota (from 6.80 ± 0.65% to 24.2 ± 0.33% of sequences), Bacteroidota (from 10.5 ± 1.22% to 18.7 ± 0.66% of sequences), Proteobacteria (from 1.10 ± 0.02% to 5.31 ± 0.36% of sequences), and Actinobacteriota (from 1.44 ± 0.21% to 1.34 ± 0.17% of sequences). The abrupt diet change impacted the relative abundances of 4 fecal bacterial phyla (Figure 6; Supplementary File S2), with the relative abundances of Bacteroidota, Fusobacteriota, and Proteobacteria being increased (*p* < 0.001) and the relative abundance of Firmicutes being decreased (*p* < 0.0001) by diet change. The abrupt diet change impacted the relative abundances of ~40 fecal bacterial genera and ~50 fecal bacterial species (Figure 7; Supplementary File S2). Fecal bacterial relative abundances were not greatly impacted by *B. coagulans* supplementation. Fecal *Bacteroides coprocola* relative abundance was lower (*p* < 0.05) in dogs supplemented with the high *B. coagulans* dose compared with the placebo. Also, fecal *Prevotella_9* relative abundance was higher (*p* < 0.01) in dogs consuming the low *B. coagulans* dose compared to those consuming the high dose.

## 4. Discussion

In the present study, an abrupt dietary transition was proposed as a model to induce disruption, or dysbiosis, to the GI microbiome of dogs, hypothesizing that dogs supplemented with a *B. coagulans* probiotic would demonstrate an enhanced capacity to adjust to the new diet due to a more stable GI microbiome. To test this, fecal characteristics and microbiota populations were evaluated in dogs supplemented with two dosage levels of *B. coagulans* or a placebo following an abrupt diet change. Overall, our findings validate that an abrupt transition from extruded kibble to canned diet substantially shifts the composition of the fecal microbiota, negatively impacting stool quality. Although *B. coagulans* did not appear to mitigate shifts in fecal consistency or microbiota populations during the diet transition, the low-dose *B. coagulans* (5 × 10^8^ CFU/d) treatment tended to improve fecal scores and modulated some fecal microbial taxa compared to dogs supplemented with the high dose (2.5 × 10^9^ CFU/d) or the placebo.

After 28 days, dogs were abruptly transitioned from a low-moisture extruded kibble comprised of ground yellow corn, poultry and porcine meal, wheat middlings, and poultry fat to a high-moisture canned diet formulated with chicken, water, meat by-products, animal liver, brewers rice, and wheat flour. Proximate analysis of the commercial diets showed that the canned diet contained more protein (42.1% vs. 22.5%, DM basis) and fat (29.7% vs. 16.1%, DM basis) than the kibble diet, while TDF content was similar (kibble: 18.1% DM basis; canned: 16.1% DM basis). The nitrogen-free extract (NFE) of the kibble diet (35.3%, DM basis) was much higher than that of the canned diet (1.7%, DM basis). Although nutrient digestibility was not measured in this study, these macronutrient compositions suggest that the kibble diet provided proportionally more starch-based substrate, while the canned diet provided proportionally more protein- and lipid-based substrate to the colon. The proportion of available carbohydrate to protein largely dictates substrate utilization by the GI microbiota [7,44], and the availability of nondigestible carbohydrates generally reduces protein fermentation in dogs [45,46]. Although protein and lipid digestion are efficient in the small intestine, canine fecal microbes are still influenced by diets high in fat [47,48] and protein [44,46,49], indicating that these substrates reach the colon when provided in excess. One of the primary ways by which GI microbiota contribute to host health is through metabolism of dietary components that the host digestive system cannot process on its own, leading to changes in the colonic environment and microbial ecosystem that further influence host health [50,51].

In the current study, diet change significantly altered fecal characteristics, with dogs consuming the canned diet having lower fecal DM percentage and greater fecal scores (i.e., looser stools). Diet transition is commonly associated with GI distress in dogs, and unabsorbed substrates reaching the colon can stimulate osmotic diarrhea [52]. Transitioning to a canned diet also increased fecal pH, indicating that more protein substrate was reaching the colon and facilitating proteolytic fermentation. Proteolytic enzymes work best at a neutral pH [53,54], facilitating the production of detrimental proteolytic metabolites, such as polyamines, that enhance bacterial pathogenicity and host susceptibility to infection [55,56,57]. Although fecal metabolites were not measured in the current study, the changes observed in fecal consistency and pH are consistent with previous studies evaluating diet change in dogs, where diets higher in protein increased fecal scores and moisture while increasing fecal pH and/or protein fermentation product concentrations [8,45].

The composition of the GI microbiota was significantly altered by diet change, where a few alpha diversity measures (Observed features, Chao1, and Faith’s PD) were reduced, while others (Simpson Index; Inverse Simpson Index) were increased or tended to increase (Shannon Diversity). These results indicate that as dogs transitioned from the kibble to canned diets, there was a marked reduction in the total number of observed taxa and phylogenetic diversity (richness) while the distribution of species (evenness) increased or remained stable. Similar results were reported in dogs transitioned at baseline from a commercial diet to either a high-carbohydrate, low-protein diet (49.4% protein, 10.9% carbohydrate) or a low-protein, high-carbohydrate diet (25.5% protein, 38.8% carbohydrate), where bacterial diversity within fecal samples had increased evenness, but not richness, compared to samples collected at baseline [44]. Although reduced richness with stable evenness suggests a narrowing of taxonomic diversity, the maintenance of community evenness may indicate functional redundancy among remaining taxa, thereby preserving overall ecosystem stability. Beta diversity, which examines the difference in microbial composition between samples, demonstrated distinct clustering of microbial communities between dogs consuming extruded kibble and canned diets, attributed to differences in macronutrient composition. Alpha and beta diversity remained largely stable between treatment groups, although phylogenetic diversity was greater in fecal samples from dogs supplemented with the low-dose *B. coagulans* treatment than those receiving the placebo, indicating a more diverse microbial ecosystem. Although it may be inferred that a more diverse community of microbes exhibits a wider range of functional capabilities, alpha diversity is a broad measure that only provides a starting point for comparison between treatment groups and the ecological significance of these differences [58]. While time and treatment independently influenced microbial diversity, there was no combined effect for any index, suggesting that *B. coagulans* did not modify the response of the microbial community to the diet change.

Shifts in fecal bacterial diversity were accompanied by compositional changes to the relative abundance of the four major bacterial phyla present in dogs [59,60,61], where Firmicutes were reduced and Bacteroidota, Fusobacteriota, and Proteobacteria were enriched after transition to the canned diet. Within these phyla, several key taxa involved in fiber degradation and SCFA production were lower (e.g., *Bifidobacterium* spp., *Faecalibacterium* spp., *Blautia* spp., *Megamonas* spp., *Prevotella* spp., and *Turicibacter* spp.), while some fat- and protein-metabolizing bacteria were greater (e.g., *Fusobacterium* spp., *Clostridium* spp., *Bacteroides* spp., and *Sutterella* spp.) in animals fed the canned diet than those fed the kibble diet. In healthy dogs, these changes signal a reduction in microbial taxa involved in saccharolytic fermentation and enrichment of amino acid-fermenting bacteria as macronutrient composition shifts from a higher-carbohydrate/lower-protein diet to a higher-protein/lower-carbohydrate diet [62,63]. Similarly, dogs fed raw meat-based diets have been reported to exhibit decreased relative abundances of Firmicutes and Bacteroidota and increased relative abundances of Proteobacteria, Fusobacteriota, and proteolytic genera within these phyla [64,65]. The relative abundance of Bacteroidota increased following diet transition in the current study, which may be attributed to diet-induced changes at the genus level within *Prevotella* and *Bacteroides.* Before the diet transition, the average relative abundance rates of *Bacteroides* and *Prevotella_9* among treatment groups were comparable (4.5% and 3.1% of sequences, respectively), together comprising the highest relative abundance within the phylum Bacteroidota. Although the relative abundance of *Prevotella_9* decreased to 0.67% of sequences after diet transition, *Bacteroides* increased nearly 3-fold to 13.2% of sequences, augmenting the relative abundance of taxa within the phylum Bacteroidota. The ratio of *Bacteroides* to *Prevotella* is influenced by diet in both humans and canines, with a higher relative abundance of *Prevotella* observed in high-carbohydrate diets, whereas a greater relative abundance of *Bacteroides* is linked with high-protein and high-fat diets [6,7,8,44,47,66,67,68].

Interestingly, fecal abundances of *Faecalibacterium* and *E. coli* measured by qPCR were greater in dogs supplemented with the low dose of *B. coagulans* than those in the control group, regardless of diet. *Faecalibacterium* spp. are associated with intestinal health through production of SCFA [69,70] and anti-inflammatory peptides [71,72], with populations often decreased in dogs with GI disease [73,74,75]. Previously, fecal samples of healthy dogs fed *B. coagulans* GBI-30, 6086 (5 × 10^8^ CFU/d) were enriched with *Faecalibacterium* compared with placebo-supplemented dogs [1]. Similarly, elderly men and women supplemented with *B. coagulans* GBI-30, 6086 (1 × 10^9^ CFU/d) had greater populations of *F. prausnitzii* compared to those consuming the placebo [27]. *B. coagulans* supplementation may increase fecal *Faecalibacterium* abundance via metabolic cross-feeding, where lactate and acetate produced by *B. coagulans* can serve as substrate for *Faecalibacterium* activity and subsequent production of butyrate [70,76]. The observed increase in fecal *E. coli* abundance in dogs supplemented with the low dose of *B. coagulans* was not anticipated, although this result may reflect ecological adjustments rather than dysbiosis [43]. *Bacillus* activity can alter intestinal conditions, such as pH and metabolite availability, in ways that transiently favor facultative anaerobes like *E. coli* [77]. Additionally, shifts in microbial competition or reductions in other taxa following probiotic administration could result in a relative increase in *E. coli* abundance. As the detected levels remained within the healthy reference range for dogs [43,73,78], this change likely represents a benign community rebalancing rather than an adverse effect.

The recommended dosage of *B. coagulans* GBI-30, 6086 for humans ranges from 1.0 × 10^8^ CFU/d to 3.0 × 10^9^ CFU/d [79]. This strain has also been evaluated in healthy adult dogs at doses between 1 × 10^6^ CFU/d and 1 × 10^9^ CFU/d, with no adverse effects reported on fecal consistency, fecal moisture, or defecation frequency. Notably, significant positive treatment effects were observed at the higher end of this dose range at 1.3 × 10^9^ CFU/d [30]. Accordingly, the present study employed two graded inclusion levels of *B. coagulans* GBI-30, 6086, designed to fall within a range previously shown to be both safe and functionally beneficial. Although *B. coagulans* supplementation had minimal effects on alleviating diet-induced dysbiosis, the low dose demonstrated modest improvements to stool quality and fecal *Faecalibacterium* abundance compared with the high dose or placebo. The observed dose-dependent effects could be indicative of a hormetic response, characterized by low-dose stimulation and high-dose inhibition, ultimately promoting adaptive stress responses that enhance host resilience and health [80]. Supplementation with the high dose of *B. coagulans* may also inadvertently inhibit certain commensal taxa or modulate the gut environment in ways that alter microbial competition and metabolic activity, warranting further investigation of host–microbe interactions and microbial functional dynamics across a broader dosage range.

Host–microbe interactions are highly complex and dependent on substrate availability as well as intestinal osmolarity, pH, and transit time [50]. As microbial species, probiotic microorganisms are also subject to these factors, potentially confounding the effects observed during diet change. Furthermore, as spore-forming bacteria, *Bacillus* spp. predominantly remain as spores to survive passage through the stomach [81,82], requiring germination in the duodenum into active vegetative cells to exert metabolic functions [83]. While both *Bacillus* spores and cells can modulate host immune responses [18], only active vegetative cells can secrete enzymes, vitamins, antimicrobial compounds, and other metabolites that can aid in regulating the GI microbiota and host digestion [13,84]. *Bacillus* has been shown to germinate in the presence of nutrients (e.g., amino acids and sugars) in the small intestine [85,86,87,88,89], although this process takes a minimum of 7–8 h [83,90,91], and the degree of germination cannot be accurately quantified without comparison of ileal digesta and fecal samples. Due to ethical and medical concerns associated with ileal canulation [92,93], companion animal studies are primarily limited to collecting fecal samples, which may not directly reflect microbial activity in the small intestine. Because fecal samples were used in this study, it was impossible to measure the level at which spore germination occurred and what impact that may have had in the current study.

A limitation of this study is the exclusive use of fecal samples to characterize microbial responses to probiotic supplementation and dietary change. While fecal samples provide some insight into the distal colonic microbiota, they do not fully reflect microbial dynamics or metabolic activity occurring in the upper gastrointestinal tract, particularly within the small intestine, where probiotic interactions and nutrient metabolism may differ substantially. Therefore, the results presented here should be interpreted as representative of the distal colonic ecosystem. Future studies incorporating samples from multiple intestinal sites or employing indirect measures of small intestinal activity would provide a more comprehensive understanding of probiotic effects along the gastrointestinal tract. Furthermore, given the profound impact of diet on stool quality and the GI microbiome, future studies evaluating the effects of probiotics in dogs undergoing diet change should aim to measure intestinal transit time and perhaps defecation frequency. The speed at which food moves through the digestive system determines how long probiotic bacteria are exposed to the gut environment and germination signals, affecting the ability for *Bacillus* spp. and other taxa to colonize and exert beneficial effects [94,95]. Collecting fecal samples at multiple time points each day may also provide additional insights, as fecal microbes and metabolites fluctuate throughout the day [96].

## 5. Conclusions

In conclusion, the change from a kibble to a canned diet affected stool characteristics, altered microbial diversity, and significantly shifted the composition of the fecal microbiota. Supplementation with *B. coagulans* did not affect fecal characteristics, but had mild effects on the fecal microbiota, with one measure suggesting increased phylogenetic diversity and altered abundance of a few fecal microbial taxa. Surprisingly, supplementation with the lower dose of *B. coagulans* (5 × 10^8^ CFU/d) had a greater influence on fecal microbial populations compared to the higher dose (2.5 × 10^9^ CFU/d), although the mechanisms underlying this dose response are unclear and warrant further investigation. Additional research is needed to investigate optimal inclusion levels in pet foods, as well as testing the effects of *B. coagulans* in spore and vegetative cell form to determine whether that impacts their activity in the GI tract.

## Figures and Tables

**Table 1:** Analyzed chemical and energy composition of the commercial diets fed to *Bacillus coagulans*-supplemented dogs.

Analyzed Composition	Kibble Diet ^1^	Canned Diet ^2^
Dry matter (DM), %	94.05	23.15
-- %, DM --		
Ash	8.06	10.37
Crude protein	22.47	42.07
Acid-hydrolyzed fat	16.07	29.72
Total dietary fiber	18.08	16.10
Insoluble fiber	15.58	11.82
Soluble fiber	2.50	4.28
Gross energy, kcal/g as-is	4.71	1.35
Gross energy, kcal/g DM	5.01	5.83
Calculated ME, kcal/g ^3^ as-is	3.19	0.94
Calculated ME, kcal/g ^3^ DM	3.39	4.06

^1^ Best Dog 21/12 (Mid-South Feeds, Inc., Alma, GA, USA), ingredients: ground yellow corn, poultry & porcine meal, wheat middlings, poultry fat (preserved with BHA), salt, calcium propionate, potassium chloride, artificial garlic flavoring, calcium carbonate, vitamin E (as D-alpha tocopheryl acetate), riboflavin supplement, niacin supplement, biotin, calcium pantothenate, vitamin A supplement, menadione sodium bisulfite complex (source of vitamin K activity), thiamine mononitrate (source of vitamin B1), pyridoxine hydrochloride (source of vitamin B6), vitamin B12 supplement, vitamin D3 supplement, ferrous sulfate, zinc sulfate, zinc oxide, manganese sulfate, copper sulfate, sodium selenite, calcium iodate, cobalt carbonate, folic acid, mineral oil. ^2^ Pedigree Chopped Ground Dinner with Chicken (Mars Petcare US, Franklin, TN, USA), ingredients: chicken, sufficient water for processing, meat by-products, animal liver, brewers rice, wheat flour, minerals (potassium chloride, magnesium proteinate, zinc sulfate, selenium, copper proteinate, manganese sulfate, copper sulfate, potassium iodide), carrageenan, sodium tripolyphosphate, dried yam, xanthan gum, vitamins (choline chloride, vitamin E supplement, thiamine mononitrate, calcium pantothenate, biotin, riboflavin supplement, vitamin A supplement, vitamin D3 supplement, vitamin B12 supplement), natural flavor, guar gum, yellow #6, yellow #5. ^3^ Metabolizable energy (ME, kcal/g) = (3.5 kcal/g × CP %) + (8.5 kcal/g × acid-hydrolyzed fat %) + (3.5 kcal/g × nitrogen-free extract %); nitrogen-free extract (%) = 100% − (CP % + acid-hydrolyzed fat % + TDF % + ash %).

## Data Availability

The sequence data generated from this study will be available at the NCBI Sequence Read Archive (http://www.ncbi.nlm.nih.gov/sra, 3 April 2025) under accession number SUB15233007 and BioProject PRJNA1246338 after 3 April 2026.

## Supplementary Materials

The following supporting information can be downloaded at: https://www.mdpi.com/article/10.3390/microorganisms13112462/s1, Supplementary File S1: Fresh fecal characteristics and bacterial abundance (log DNA/gram feces) of *Bacillus coagulans* GBI-30, 6086-supplemented dogs before and after an abrupt 14-day diet change; Supplementary File S2: Predominant fecal bacterial abundances (relative abundance, %) of *Bacillus coagulans* GBI-30, 6086-supplemented dogs before and after an abrupt 14-day diet change.

## Abbreviations

The following abbreviations are used in this manuscript:

AHF	acid-hydrolyzed fat
CFU	colony-forming units
CP	crude protein
DM	dry matter
GI	gastrointestinal
NFE	nitrogen-free extract
OM	organic matter
PD	phylogenetic diversity
qPCR	quantitative polymerase chain reaction
SCFA	short-chain fatty acids
TDF	total dietary fiber
