# Supplementary material for: Effects of Bacillus coagulans (GBI-30, 6086) Supplementation on the Fecal Characteristics and Microbiota of Healthy Adult Dogs Subjected to an Abrupt Diet Change
Source: Microorganisms. 2025 Oct 28;13(11):2462. doi: 10.3390/microorganisms13112462 (PMC12654280; doi:10.3390/microorganisms13112462)
Supplement: Supplementary file 1 [file microorganisms-13-02462-s001.zip › Wilson_KerryDogStudy2_DietTransition(File S2)_Microorganisms.pdf]

Supplementary File S2. Predominant fecal bacterial abundances (relative abundance, %) of *Bacillus coagulans* GBI-30, 6086-supplemented dogs before and after an abrupt 14-day diet change.

Taxa			Before (Day 0)			Day 2			Day 6			Day 10			Day 14			SEM[1]	p-value	
**Phylum**	**Genus**	**Species**	**Control**	**Low**	**High**	**Control**	**Low**	**High**	**Control**	**Low**	**High**	**Control**	**Low**	**High**	**Control**	**Low**	**High**		**Treatment**	**Time**
Actinobacteriota			1.24	1.72	1.35	1.33	1.26	1.31	1.48	1.41	1.34	1.24	1.47	1.59	1.31	1.16	1.56	0.23	0.8955	0.5344
	Bifidobacterium		0.53	0.59	0.58	0.01	0.02	0.02	0.01	0.02	0.00	0.01	0.04	0.00	0.01	0.00	-0.01	0.13	0.7360	**<0.0001**
	Bifidobacterium	*pseudolongum*	0.09	0.40	0.27	0.00	0.01	0.01	0.00	0.02	0.00	0.00	0.03	0.00	0.00	0.00	0.00	0.10	0.6148	**<0.0001**
	Bifidobacterium	*animalis*	0.30	0.08	0.24	0.01	0.00	0.00	0.00	0.00	0.00	0.01	0.01	0.00	0.00	0.00	0.00	0.06	0.2909	**0.0012**
	Bifidobacterium	*pullorum*	0.14	0.12	0.06	0.00	0.00	0.00	0.00	0.00	0.00	0.00	0.00	0.00	0.00	0.00	0.00	0.03	0.6429	**<0.0001**
	Collinsella		0.63	1.03	0.69	1.11	1.01	1.09	1.20	1.17	1.14	0.96	1.20	1.32	1.04	0.92	1.30	0.18	0.7765	**0.0005**
	Collinsella	Unclassified	0.06	0.08	0.07	0.15	0.12	0.14	0.17	0.12	0.15	0.12	0.14	0.17	0.11	0.10	0.16	0.02	0.7922	**<0.0001**
	Collinsella	*intestinalis*	0.56	0.93	0.61	0.90	0.85	0.92	0.97	0.98	0.95	0.81	0.99	1.12	0.88	0.78	1.08	0.15	0.7245	**0.0029**
	Slackia		0.08	0.11	0.08	0.21	0.23	0.21	0.27	0.22	0.20	0.26	0.23	0.28	0.26	0.23	0.26	0.03	0.9453	**<0.0001**
	Slackia	*faecicanis*	0.04	0.07	0.05	0.18	0.20	0.19	0.27	0.21	0.20	0.26	0.22	0.27	0.26	0.23	0.26	0.03	0.9402	**<0.0001**
Bacteroidota			8.80	11.67	10.93	15.26	14.23	16.37	12.53	17.32	13.89	17.04	14.30	13.17	19.10	19.15	17.73	2.11	0.8798	**0.0002**
	Alloprevotella		2.22	1.86	1.94	2.54	2.31	3.50	2.52	4.09	2.34	3.45	2.53	1.71	3.62	3.62	2.96	0.64	0.4507	**0.0389**
	Alloprevotella	Unclassified	2.22	1.86	1.94	2.54	2.31	3.50	2.52	4.09	2.34	3.45	2.53	1.71	3.62	3.62	2.96	0.64	0.4507	**0.0389**
	Bacteroides		3.25	5.14	5.06	11.41	10.18	11.36	8.98	11.27	10.52	11.87	10.16	10.27	13.85	12.92	12.96	1.37	0.9686	**<0.0001**
	Bacteroides	*coprocola*	0.12	0.13	0.10	0.13	0.04	0.24	0.12	0.22	0.21	0.27	0.16	0.19	0.40	0.20	0.23	0.12	**0.0148**	**0.0067**
	Bacteroides	*plebeius*	0.26	0.32	0.28	0.30	0.34	0.16	0.05	0.10	0.00	0.01	0.02	0.00	0.00	0.01	0.00	0.07	0.0807	**<0.0001**
	Bacteroides	Unclassified	2.82	4.57	4.60	10.98	9.81	10.96	8.80	10.94	10.31	11.59	9.96	10.08	13.44	12.71	12.73	1.33	0.9703	**<0.0001**
	Parabacteroides		0.14	0.15	0.15	0.15	0.13	0.21	0.03	0.15	0.07	0.07	0.11	0.03	0.08	0.15	0.07	0.04	0.2985	**0.0040**
	Parabacteroides	Unclassified	0.13	0.14	0.14	0.14	0.12	0.20	0.02	0.13	0.07	0.05	0.10	0.03	0.07	0.13	0.06	0.04	0.3558	**0.0011**
	Prevotella_9		2.58	3.69	3.04	0.25	0.63	0.32	0.17	0.76	0.04	0.59	0.43	0.03	0.45	1.21	0.35	0.50	**0.0013**	**<0.0001**
	Prevotella_9	Unclassified	2.55	3.62	2.99	0.25	0.62	0.32	0.17	0.76	0.04	0.59	0.43	0.03	0.45	1.21	0.35	0.50	**0.0013**	**<0.0001**
	Prevotellaceae Ga6A1 group		0.44	0.41	0.46	0.61	0.65	0.56	0.43	0.43	0.45	0.62	0.55	0.47	0.54	0.67	0.61	0.14	0.7791	*0.0960*
	Prevotellaceae Ga6A1 group	Unclassified	0.44	0.41	0.46	0.61	0.65	0.56	0.43	0.43	0.45	0.62	0.55	0.47	0.54	0.67	0.61	0.14	0.7791	0.0960
	Rikenellaceae RC9 gut group		0.08	0.17	0.11	0.08	0.07	0.10	0.05	0.13	0.06	0.05	0.09	0.04	0.07	0.07	0.06	0.04	0.8939	0.9215
	Rikenellaceae RC9 gut group	Unclassified	0.08	0.17	0.11	0.08	0.07	0.10	0.05	0.13	0.06	0.05	0.09	0.04	0.07	0.07	0.06	0.04	0.8939	0.9215
	Unclassified		0.10	0.24	0.17	0.23	0.26	0.32	0.32	0.50	0.43	0.36	0.34	0.64	0.47	0.51	0.73	0.17	0.6534	**<0.0001**

	Unclassified	Unclassified	0.10	0.24	0.17	0.23	0.26	0.32	0.32	0.50	0.43	0.36	0.34	0.64	0.47	0.51	0.73	0.17	0.6534	**<0.0001**
Firmicutes			66.21	58.59	62.67	49.42	50.96	47.00	54.02	48.39	51.78	45.96	48.97	50.23	42.09	43.24	44.75	3.32	0.7548	**<0.0001**
	[Eubacterium] brachy group		0.25	0.34	0.28	2.08	2.27	2.36	1.81	1.61	1.78	1.23	1.56	1.37	1.00	1.00	1.32	0.22	0.7238	**<0.0001**
	[Eubacterium] brachy group	Unclassified	0.25	0.34	0.28	2.08	2.27	2.36	1.81	1.61	1.78	1.23	1.56	1.37	1.00	1.00	1.32	0.22	0.7238	**<0.0001**
	[Eubacterium] nodatum group		0.01	0.20	0.03	0.15	0.31	0.12	0.03	0.25	0.03	0.14	0.23	0.06	0.15	0.24	0.03	0.10	0.3308	0.4860
	[Eubacterium] nodatum group	Unclassified	0.01	0.20	0.03	0.15	0.31	0.12	0.03	0.25	0.03	0.14	0.23	0.06	0.15	0.24	0.03	0.10	0.3308	0.4860
	[Ruminococcus] gauvreauii group	Unclassified	0.25	0.28	0.27	0.04	0.05	0.04	0.04	0.04	0.05	0.03	0.04	0.04	0.03	0.04	0.04	0.03	0.6321	**<0.0001**
	[Ruminococcus] gnavus group		0.08	0.18	0.06	0.24	0.23	0.10	0.59	0.44	0.38	0.29	0.36	0.54	0.22	0.46	0.73	0.14	0.8840	**<0.0001**
	[Ruminococcus] gnavus group	Unclassified	0.03	0.06	0.02	0.15	0.18	0.04	0.38	0.29	0.29	0.18	0.21	0.35	0.13	0.26	0.49	0.13	0.9007	**<0.0001**
	[Ruminococcus] gnavus group	*gnavus*	0.05	0.12	0.04	0.08	0.05	0.06	0.21	0.16	0.09	0.11	0.15	0.20	0.09	0.21	0.23	0.07	0.6024	0.8973
	[Ruminococcus] torques group		2.06	2.25	1.66	1.92	1.93	1.70	1.95	1.65	1.85	1.69	2.22	2.14	1.56	1.56	1.75	0.29	0.7870	0.1830
	[Ruminococcus] torques group	Unclassified	2.06	2.25	1.66	1.92	1.93	1.70	1.95	1.65	1.85	1.69	2.22	2.14	1.56	1.56	1.75	0.29	0.7870	0.1830
	Allobaculum		1.00	1.83	2.23	2.54	2.80	2.94	2.26	2.65	2.21	2.98	2.37	2.42	3.10	2.53	2.22	0.53	0.8088	**<0.0001**
	Allobaculum	*stercoricanis*	1.00	0.90	0.71	2.54	2.79	2.36	2.27	2.60	2.07	2.99	2.21	2.09	3.11	2.53	2.04	0.34	0.0944	**<0.0001**
	Allobaculum	Unclassified	0.00	0.93	1.52	0.00	0.01	0.58	0.00	0.04	0.14	-0.01	0.20	0.33	0.00	0.00	0.18	0.47	0.0994	0.4324
	Blautia		10.16	9.45	9.65	4.11	3.50	4.04	4.15	3.96	4.65	3.09	4.33	4.41	2.88	3.82	3.58	0.82	0.8400	**<0.0001**
	Blautia	*glucerasea*	0.43	0.47	0.40	0.11	0.08	0.08	0.08	0.09	0.09	0.06	0.08	0.07	0.04	0.07	0.05	0.05	0.8630	**<0.0001**
	Blautia	*hansenii*	1.28	1.27	1.17	0.94	0.93	1.08	1.42	1.26	1.14	1.26	1.69	1.46	0.99	1.09	1.23	0.26	0.9417	0.0503
	Blautia	Unclassified	8.41	7.19	8.04	3.05	2.46	2.87	2.66	2.60	3.42	1.77	2.57	2.87	1.85	2.66	2.29	0.68	0.8425	**<0.0001**
	Candidatus stoquefichus		0.05	0.06	0.09	0.35	0.46	0.34	0.49	0.43	0.49	0.53	0.46	0.50	0.69	0.48	0.30	0.09	0.4298	**<0.0001**
	Candidatus stoquefichus	Unclassified	0.05	0.06	0.09	0.35	0.46	0.34	0.49	0.43	0.49	0.53	0.46	0.50	0.69	0.48	0.30	0.09	0.4298	**<0.0001**
	Catenibacterium		1.03	0.70	1.34	0.14	0.15	0.26	0.06	0.28	0.08	0.07	0.02	0.05	0.03	0.07	0.29	0.18	0.4275	**<0.0001**
	Catenibacterium	*mitsuokai*	1.03	0.70	1.34	0.14	0.15	0.26	0.06	0.28	0.08	0.07	0.02	0.05	0.03	0.07	0.29	0.18	0.4275	**<0.0001**
	Clostridium sensu stricto 1		0.39	0.22	0.06	2.12	2.63	2.53	5.60	2.43	4.47	3.19	3.89	5.10	1.97	2.49	3.07	0.71	0.8557	**<0.0001**
	Clostridium sensu stricto 1	*perfringens*	0.30	0.04	0.04	1.93	2.45	2.28	4.73	2.09	3.73	2.83	3.27	4.54	1.62	2.06	2.93	0.62	0.8417	**<0.0001**
	Clostridium sensu stricto 1	*colicanis*	0.00	0.00	0.00	0.18	0.16	0.22	0.80	0.30	0.70	0.32	0.59	0.50	0.33	0.38	0.10	0.24	0.4444	**<0.0001**
	Enterococcus		2.01	1.47	0.76	0.10	0.18	0.16	0.01	0.07	0.01	0.04	0.03	0.11	0.01	0.02	0.01	0.33	0.4678	**<0.0001**
	Enterococcus	*cecorum*	2.01	1.47	0.76	0.10	0.18	0.16	0.01	0.07	0.01	0.04	0.03	0.11	0.01	0.02	0.01	0.33	0.4678	**<0.0001**
	Erysipelatoclostridium		0.46	0.53	0.35	0.10	0.09	0.07	0.05	0.05	0.04	0.04	0.07	0.05	0.03	0.03	0.06	0.06	0.3964	**<0.0001**
	Erysipelatoclostridium	Unclassified	0.46	0.53	0.35	0.10	0.09	0.07	0.05	0.05	0.04	0.04	0.07	0.05	0.03	0.03	0.06	0.06	0.3964	**<0.0001**
	Erysipelotrichaceae UCG-003		0.70	0.93	0.93	0.14	0.05	0.11	0.03	0.05	0.06	0.01	0.04	0.14	0.00	0.03	0.16	0.14	0.2550	**<0.0001**

Erysipelotrichaceae UCG-003	*bacterium*	0.70	0.93	0.93	0.14	0.05	0.11	0.03	0.05	0.06	0.01	0.04	0.14	0.00	0.03	0.16	0.14	0.2550	**<0.0001**
Faecalibacterium		0.62	0.66	0.63	0.22	0.11	0.18	0.11	0.24	0.13	0.17	0.12	0.08	0.17	0.15	0.10	0.07	0.3467	**<0.0001**
Faecalibacterium	Unclassified	0.62	0.66	0.63	0.22	0.11	0.18	0.11	0.24	0.13	0.17	0.12	0.08	0.17	0.15	0.10	0.07	0.3467	**<0.0001**
Holdemanella		1.53	1.57	1.37	0.45	0.41	0.45	0.28	0.68	0.27	0.23	0.30	0.26	0.15	0.20	0.52	0.21	0.8165	**<0.0001**
Holdemanella	Unclassified	1.53	1.57	1.37	0.45	0.41	0.45	0.28	0.68	0.27	0.23	0.30	0.26	0.15	0.20	0.52	0.21	0.8165	**<0.0001**
Lachnoclostridium		0.33	0.44	0.37	0.36	0.30	0.32	0.34	0.34	0.37	0.33	0.37	0.37	0.40	0.33	0.33	0.05	0.7236	0.6706
Lachnoclostridium	Unclassified	0.33	0.44	0.37	0.36	0.30	0.32	0.34	0.34	0.37	0.33	0.37	0.37	0.40	0.33	0.33	0.05	0.7236	0.6706
Lachnospiraceae NK4A136 group	Unclassified	0.10	0.10	0.09	0.05	0.05	0.05	0.06	0.06	0.05	0.04	0.06	0.05	0.04	0.06	0.05	0.01	0.8420	**0.0012**
Lactobacillus		1.90	1.36	3.83	0.00	0.05	0.00	0.07	0.00	-0.01	0.07	0.01	-0.01	0.07	0.00	-0.01	0.95	0.9814	**<0.0001**
Lactobacillus	*acidophilus*	1.64	1.09	2.12	0.00	0.04	0.00	0.05	0.00	0.00	0.06	0.01	0.00	0.05	0.00	0.00	0.62	0.7560	**<0.0001**
Lactobacillus	*johnsonii*	0.26	0.28	1.72	0.00	0.01	0.00	0.01	0.00	0.00	0.01	0.00	0.00	0.01	0.00	0.00	0.36	0.6460	**<0.0001**
Ligilactobacillus		12.75	7.02	6.60	0.04	0.08	0.02	0.00	0.01	0.00	0.00	0.02	0.01	0.00	0.01	0.00	1.28	0.5441	**<0.0001**
Ligilactobacillus	Unclassified	12.75	7.02	6.60	0.04	0.08	0.02	0.00	0.01	0.00	0.00	0.02	0.01	0.00	0.01	0.00	1.28	0.5441	**<0.0001**
Limosilactobacillus		0.94	0.84	1.15	0.01	0.04	0.00	0.02	0.00	0.00	0.02	0.00	0.00	0.02	0.00	0.00	0.19	0.7435	**<0.0001**
Limosilactobacillus	Unclassified	0.94	0.84	1.15	0.01	0.04	0.00	0.02	0.00	0.00	0.02	0.00	0.00	0.02	0.00	0.00	0.19	0.7435	**<0.0001**
Megamonas		2.15	1.23	1.54	0.21	0.15	0.14	0.18	0.28	0.16	0.20	0.23	0.09	0.31	0.30	0.23	0.21	0.4235	**<0.0001**
Megamonas	Unclassified	2.15	1.23	1.54	0.21	0.15	0.14	0.18	0.28	0.16	0.20	0.23	0.09	0.31	0.30	0.23	0.21	0.4235	**<0.0001**
Negativibacillus		0.21	0.36	0.24	1.12	1.30	1.40	0.93	1.25	1.22	1.10	1.37	0.72	1.25	1.39	0.95	0.24	0.5010	**<0.0001**
Negativibacillus	Unclassified	0.21	0.36	0.24	1.12	1.30	1.40	0.93	1.25	1.22	1.10	1.37	0.72	1.25	1.39	0.95	0.24	0.5010	**<0.0001**
Paeniclostridium		0.00	0.00	0.00	0.44	0.70	0.56	1.35	1.08	0.65	0.55	0.65	1.07	0.33	0.43	0.48	0.23	0.7314	**<0.0001**
Paeniclostridium	Unclassified	0.00	0.00	0.00	0.44	0.70	0.56	1.35	1.08	0.65	0.55	0.65	1.07	0.33	0.43	0.48	0.23	0.7314	**<0.0001**
Peptoclostridium		7.88	8.55	7.89	20.52	20.91	19.19	24.63	20.83	23.60	22.10	21.86	21.58	19.84	19.74	20.16	1.78	0.7704	**<0.0001**
Peptoclostridium	Unclassified	7.88	8.55	7.89	20.52	20.91	19.19	24.63	20.83	23.60	22.10	21.86	21.58	19.84	19.74	20.16	1.78	0.7704	**<0.0001**
Peptococcus		0.70	0.72	0.61	1.74	1.95	1.67	2.67	2.00	2.08	2.23	1.96	2.23	2.16	1.65	1.81	0.23	0.4486	**<0.0001**
Peptococcus	Unclassified	0.70	0.72	0.61	1.74	1.95	1.67	2.67	2.00	2.08	2.23	1.96	2.23	2.16	1.65	1.81	0.23	0.4486	**<0.0001**
Phascolarctobacterium		1.32	1.73	1.37	1.88	2.09	2.05	1.96	2.04	1.96	1.76	1.75	1.85	2.20	1.84	1.92	0.20	0.5921	**0.0005**
Phascolarctobacterium	Unclassified	1.32	1.73	1.37	1.88	2.09	2.05	1.96	2.04	1.96	1.76	1.75	1.85	2.20	1.84	1.92	0.20	0.5921	**0.0005**
Sellimonas		0.37	0.35	0.39	0.78	0.75	0.68	0.30	0.48	0.47	0.34	0.45	0.40	0.29	0.34	0.27	0.07	0.7588	**<0.0001**
Sellimonas	Unclassified	0.37	0.35	0.39	0.78	0.75	0.68	0.30	0.48	0.47	0.34	0.45	0.40	0.29	0.34	0.27	0.07	0.7588	**<0.0001**
Streptococcus		15.02	12.72	16.17	6.13	5.81	3.73	2.93	3.55	3.71	2.33	2.99	3.72	1.89	2.71	3.53	2.11	0.7479	**<0.0001**
Streptococcus	*lutetiensis*	13.20	11.52	14.44	5.95	5.58	3.64	2.87	3.49	3.64	2.18	2.95	3.68	1.85	2.67	3.50	2.09	0.5631	**<0.0001**

	Streptococcus	*alactolyticus*	1.82	1.20	1.73	0.17	0.23	0.09	0.07	0.06	0.08	0.15	0.02	0.06	0.05	0.04	0.04	0.30	0.8438	**<0.0001**
	Turicibacter		0.81	0.90	1.11	0.03	0.05	0.02	0.02	0.02	0.01	0.04	0.01	0.02	0.02	0.02	0.01	0.17	0.5396	**<0.0001**
	Turicibacter	Unclassified	0.80	0.86	1.09	0.02	0.04	0.01	0.00	0.01	0.00	0.02	0.00	0.00	-0.01	0.01	0.00	0.17	0.6628	**<0.0001**
	Tuzzerella		0.24	0.26	0.14	0.19	0.18	0.24	0.06	0.10	0.04	0.06	0.06	0.02	0.07	0.07	0.03	0.05	0.1204	**<0.0001**
	Tuzzerella	Unclassified	0.24	0.26	0.14	0.19	0.18	0.24	0.06	0.10	0.04	0.06	0.06	0.02	0.07	0.07	0.03	0.05	0.1204	**<0.0001**
	UCG-005		0.25	0.23	0.30	0.41	0.44	0.40	0.36	0.57	0.34	0.34	0.32	0.20	0.41	0.46	0.17	0.09	0.2510	0.2609
	UCG-005	Unclassified	0.25	0.23	0.30	0.41	0.44	0.40	0.36	0.57	0.34	0.34	0.32	0.20	0.41	0.46	0.17	0.09	0.2510	0.2609
	Unclassified		0.04	0.04	0.03	0.13	0.13	0.13	0.13	0.11	0.14	0.11	0.10	0.10	0.10	0.08	0.13	0.02	0.6871	**<0.0001**
	Unclassified	Unclassified	0.04	0.04	0.03	0.13	0.13	0.13	0.13	0.11	0.14	0.11	0.10	0.10	0.10	0.08	0.13	0.02	0.6871	**<0.0001**
	Unclassified		0.07	0.10	0.12	0.32	0.24	0.33	0.22	0.33	0.26	0.22	0.27	0.12	0.21	0.24	0.14	0.05	0.5603	**<0.0001**
	Unclassified	Unclassified	0.07	0.10	0.12	0.32	0.24	0.33	0.22	0.33	0.26	0.22	0.27	0.12	0.21	0.24	0.14	0.05	0.5603	**<0.0001**
	Unclassified		0.14	0.51	0.55	0.15	0.27	0.39	0.14	0.18	0.19	0.14	0.22	0.33	0.15	0.13	0.29	0.14	0.3722	0.5391
	Unclassified	Unclassified	0.14	0.51	0.55	0.15	0.27	0.39	0.14	0.18	0.19	0.14	0.22	0.33	0.15	0.13	0.29	0.14	0.3722	0.5391
Fusobacteriota			5.89	7.42	7.08	20.71	20.03	21.07	20.97	20.53	20.63	24.04	22.59	23.31	24.37	24.54	23.76	1.31	0.9769	**<0.0001**
	Fusobacterium		5.89	7.42	7.08	20.71	20.03	21.07	20.97	20.53	20.63	24.04	22.59	23.31	24.37	24.54	23.76	1.31	0.9769	**<0.0001**
	Fusobacterium	Unclassified	3.82	4.79	4.04	16.45	16.66	16.57	18.11	16.37	16.23	20.09	18.52	19.45	19.87	19.71	18.51	1.14	0.8841	**<0.0001**
	Fusobacterium	*mortiferum*	0.12	0.20	0.51	0.02	0.03	0.13	0.00	0.05	0.50	0.00	0.14	0.32	0.02	0.08	0.18	0.21	0.9158	0.6614
	Fusobacterium	*perfoetens*	1.96	2.43	2.53	4.24	3.34	4.37	2.86	4.10	3.94	3.94	4.01	3.59	4.48	4.75	5.12	0.62	0.7095	**<0.0001**
Proteobacteria			1.10	1.08	1.12	4.70	4.43	4.91	4.10	4.28	5.03	5.13	5.28	4.68	5.75	4.88	5.29	0.49	0.9373	**<0.0001**
	Escherichia-Shigella		0.16	0.19	0.34	0.25	0.30	0.27	0.22	0.19	0.13	0.12	0.43	0.38	0.09	0.07	0.04	0.11	0.7704	**0.0009**
	Escherichia-Shigella	*coli*	0.09	0.15	0.19	0.17	0.18	0.13	0.13	0.12	0.08	0.06	0.20	0.23	0.05	0.05	0.02	0.06	0.6830	**0.0011**
	Parasutterella		0.08	0.08	0.08	0.61	0.66	0.80	0.71	0.66	0.84	0.81	0.80	0.71	1.01	0.68	0.83	0.14	0.9026	**<0.0001**
	Parasutterella	*secunda*	0.08	0.08	0.08	0.61	0.66	0.80	0.71	0.66	0.84	0.81	0.80	0.71	1.01	0.68	0.83	0.14	0.9026	**<0.0001**
	Sutterella		0.86	0.80	0.70	3.84	3.46	3.85	3.17	3.43	4.05	4.21	4.06	3.60	4.66	4.12	4.42	0.42	0.8923	**<0.0001**
	Sutterella	Unclassified	0.86	0.80	0.70	3.84	3.46	3.85	3.17	3.43	4.05	4.21	4.06	3.60	4.66	4.12	4.42	0.42	0.8923	**<0.0001**

[1]SEM = pooled standard errors of the means.
