# Supplementary material for: Effects of Bacillus coagulans (GBI-30, 6086) Supplementation on the Fecal Characteristics and Microbiota of Healthy Adult Dogs Subjected to an Abrupt Diet Change
Source: Microorganisms. 2025 Oct 28;13(11):2462. doi: 10.3390/microorganisms13112462 (PMC12654280; doi:10.3390/microorganisms13112462)
Supplement: Supplementary file 1 [file microorganisms-13-02462-s001.zip › Wilson_KerryDogStudy2_DietTransition(File S1)_Microorganisms.pdf]

Supplementary File S1. Fresh fecal characteristics and bacterial abundance (log DNA/gram feces) of *Bacillus coagulans* GBI-30, 6086-supplemented dogs before and after an abrupt 14-day diet change.

Item		Before (Day 0)			Day 2			Day 6			Day 10			Day 14			SEM[2]	p-value		
	Reference Range[1]	Control	Low	High	Control	Low	High	Control	Low	High	Control	Low	High	Control	Low	High		Treatment	Day	Treatment*Day
Fecal score		3.2	2.8	2.8	3.5	3.3	3.5	3.5	3.3	3.5	3.6	3.5	3.5	3.3	3.3	3.4	0.12	*0.0883*	**<0.0001**	0.5526
pH		6.19	5.9	5.87	6.62	6.53	6.69	6.69	6.67	6.72	6.63	6.65	6.7	6.57	6.51	6.56	0.1	0.337	**<0.0001**	0.5604
Dry matter, %		27.52	28.29	28.91	21.93	20.99	21.27	21.25	21.45	19.71	20.78	19.7	19.79	22.36	22.19	20.36	0.81	0.2734	**<0.0001**	0.3706
--- Log DNA ---																				
Total bacteria	---	10.6	10.6	10.6	10.6	10.6	10.5	10.6	10.6	10.6	10.6	10.6	10.6	10.4	10.6	10.6	0.04	0.5222	0.8644	NA[4]
Bacteroides	3.2-7.2	5	5.3	5.2	4.9	4.9	5.1	4.7	5.1	4.9	4.9	5	4.8	4.9	5.2	5	0.17	*0.0895*	0.1116	NA
Bifidobacterium	1.3-6.4	5.1	5.9	5.5	4.2	4.3	4.3	3.3	4.2	3.8	3.6	3.8	3.5	3.6	3.3	3.3	0.32	*0.0600*	**<0.0001**	0.5260
Blautia	9.5-11.0	9.7	9.8	9.8	9.6	9.4	9.5	9.5	9.5	9.6	9.7	9.7	9.6	9.5	9.7	9.7	0.1	0.7452	**0.0068**	NA
Clostridium hiranonis	5.1-7.1	5.5	5.6	5.6	6.4	6.3	6.3	6.3	6.3	6.4	6.3	6.4	6.3	6.3	6.4	6.4	0.07	0.9195	**<0.0001**	NA
Collinsella	---	13.8	13.9	13.9	14.2	14.1	14.1	14.1	14.3	14.3	14.3	14.3	14.2	14	13.9	14.2	0.09	0.7264	**<0.0001**	NA
Escherichia coli	0.9-8.0	5.5	5.9	5.6	5.8	5.9	5.9	5.4	5.8	5.7	5.3	5.9	5.6	5.1	5.6	5.4	0.25	**0.0088**	*0.0959*	0.9945
Faecalibacterium	3.4-8.0	4.8	5.4	5.1	4.7	4.7	4.9	4.2	4.9	4.5	4.6	4.7	4.5	4.9	5	4.9	0.15	**0.0048**	**<0.0001**	0.1934
Fusobacterium	7.0-11.3	7.6	8	7.8	8.6	8.5	8.7	8.1	8.8	8.6	8.6	8.8	8.2	8.6	9	8.7	0.21	0.1089	**<0.0001**	NA
Prevotella copri	---	13	13.4	13.2	11.5	11.8	11.6	10.5	11.7	10.7	11.3	11.4	10.1	11.2	11.9	11.2	0.3	**0.0309**	**<0.0001**	NA
Ruminococcus gnavus	---	9.4	10	9.6	10.3	9.9	10.3	10.2	10.3	10	10.6	10.4	10.4	10.5	10.6	10.6	0.2	0.9122	**<0.0001**	0.3569
Streptococcus	1.9-8.0	6.8	7	7	7.1	7	6.8	6.7	6.8	6.9	6.6	6.8	6.7	6.5	6.5	6.7	0.18	0.9723	**0.0069**	NA
Turicibacter	4.6-8.1	7.2	7.1	7.6	6.1	6.2	6.1	6.1	6	5.9	6.1	6.1	6.1	5.7	6.1	6	0.2	0.7923	**<0.0001**	0.4244

[1]Reference ranges were provided from the Texas A&M University Gastrointestinal Laboratory.
[2]SEM = pooled standard errors of the means.
[3]Fecal scores: 1 = hard, dry pellets; small hard mass; 2 = hard formed, dry stool; remains firm and soft; 3 = soft, formed and moist stool, retains shape; 4 = soft, unformed stool; assumes shape of container; 5 = watery, liquid that can be poured.
[4]NA: not normal data, requiring non-parametric statistical analysi
